# Gaze Dispersion During a Sustained-Fixation Task as a Proxy of Visual Attention in Children with ADHD

**DOI:** 10.3390/vision9030076

**Published:** 2025-09-01

**Authors:** Lionel Moiroud, Ana Moscoso, Eric Acquaviva, Alexandre Michel, Richard Delorme, Maria Pia Bucci

**Affiliations:** 1 ICAR UMR 5191, CNRS, ENS de Lyon, Site Descartes, 15 parvis René Descartes, BP 7000, 69342 Lyon Cedex 07, France; 2 Neurodiderot, INSERM, Robert Debré Hospital, University of Paris, 75019 Cedex Paris, France; 3 Child and Adolescent Psychiatry Department, Robert Debré Hospital, APHP & Université Paris Cité, 75019 Cedex Paris, France; ana.moscoso@aphp.fr (A.M.); eric.acquaviva@aphp.fr (E.A.); alexandre.michel@aphp.fr (A.M.); richard.delorme@aphp.fr (R.D.); 4 Human Genetics and Cognitive Functions, Institut Pasteur, 75015 Paris, France

**Keywords:** neurodevelopment, brain, biomarkers, endophenotype

## Abstract

Aim: The aim of this preliminary study was to explore the visual attention in children with ADHD using eye-tracking, and to identify a relevant quantitative proxy of their attentional control. Methods: Twenty-two children diagnosed with ADHD (aged 7 to 12 years) and their 24 sex-, age-matched control participants with typical development performed a visual sustained-fixation task using an eye-tracker. Fixation stability was estimated by calculating the bivariate contour ellipse area (BCEA) as a continuous index of gaze dispersion during the task. Results: Children with ADHD showed a significantly higher BCEA than control participants (*p* < 0.001), reflecting their increased gaze instability. The impairment in gaze fixation persisted even in the absence of visual distractors, suggesting intrinsic attentional dysregulation in ADHD. Conclusions: Our results provide preliminary evidence that eye-tracking coupled with BCEA analysis, provides a sensitive and non-invasive tool for quantifying visual attentional resources of children with ADHD. If replicated and extended, the increased use of gaze instability as an indicator of visual attention in children could have a major impact in clinical settings to assist clinicians. This analysis focuses on overall gaze dispersion rather than fine eye micro-movements such as microsaccades.

## 1. Introduction

Attention deficit hyperactivity disorder (ADHD) is a common neurodevelopmental disorder affecting approximately 5 to 10% of school-aged children in Western countries [1,2]. It is characterized by marked inattention, motor hyperactivity, and behavioral impulsivity, leading to significant functional impairment in in both academic and daily life settings. Children with ADHD often exhibit deficits in executive motor control, attentional regulation, and inhibitory processes [3]. These impairments may, in part, be linked to dysfunctions in multisensory integration, particularly visual and vestibular inputs [4].

Neuroimaging studies have identified structural and functional alterations in several brain regions in individuals with ADHD, including the cerebellar vermis, thalamus, and cerebellum [5,6,7], as well as abnormalities in frontostriatal circuits that are crucial for motor inhibition and executive control [8]. More recently, resting-state fMRI studies have revealed atypical developmental trajectories in areas such as the left middle temporal gyrus, inferior frontal gyrus, and insula in children with ADHD [9]. These cortical and subcortical networks are also involved in the regulation of eye movements [10], which may explain why impaired eye movement performance has been consistently reported in this population.

Specifically, children with ADHD show increased gaze instability during fixation tasks, an increase in involuntary saccades, and more errors in antisaccade tasks, reflecting a dysregulation of inhibitory control [2,11,12,13,14]. Notably, these impairments persist even in the absence of distractors, suggesting an intrinsic dysregulation of attentional processes [15,16]. Among oculomotor paradigms, the fixation task is both simple and demanding, requiring sustained attentional engagement. It involves a distributed neural network including the frontal eye fields [17], the posterior parietal cortex [18], and subcortical structures such as the superior colliculus [19]. The introduction of visual distractors reduces the effectiveness of the fixation system and increases the difficulty of maintaining gaze on the initial target [20].

In this context, eye-tracking technology offers an objective and non-invasive means to assess attentional and inhibitory functions. In particular, the ability to maintain stable visual fixation is considered a fundamental behavioral marker of these cognitive processes [21]. The shape and structure of the fixation stimulus can influence gaze stability, as shown by Thaler et al. (2013), who compared different types of targets and demonstrated that the presence or absence of a central cue significantly altered fixation performance [22]. The Bivariate Contour Ellipse Area (BCEA) is a quantitative metric of fixation stability that captures the dispersion of gaze around a fixation target [23]. Although historically used in microperimetry in retinal analysis [24], BCEA has yet to be widely applied in the context of neurodevelopmental disorders such as ADHD.

Importantly, fixation-instability ADHD could also represent an intermediate heritable phenotype, as suggested by genetic studies in the general population [25]. These findings highlight the potential of eye tracking as a valuable tool for clinical phenotyping.

In this context, the present preliminary pilot study introduces a novel, passive, and calibrated eye-tracking protocol designed to measure fixation stability in children with ADHD, compared to typically developing peers. Our objective is to assess visual attention by computing BCEA as a continuous and sensitive index of gaze dispersion during a simple fixation task. We aim to measure the fixation stability by calculating the bivariate contour ellipse area (BCEA) as a continuous index of gaze dispersion during the task, in ADHD. To our knowledge, no prior study has systematically applied this approach in children with ADHD.

## 2. Methods

### 2.1. Subjects

Twenty-four children with ADHD aged between 7 and 12 years (8.7 ± 0.96 y.o.) and naïve of any psychotropic drug were enrolled in the study. Children with ADHD were recruited at the Child and Adolescent Psychiatry Department, Robert Debré Hospital (Paris, France) and their diagnosis was based on DSM-5R criteria [1] (and made by experts in the field. The diagnosis of ADHD and the main axis I comorbidities were carried out using the Schedule for Affective Disorders and Schizophrenia for School-Age Children-Present interview (Kiddie-SADS-EP) [25]. The ADHD Rating Scale—parental version (ADHD-RS) was used to estimate the severity of the ADHD-related symptoms. The Wechsler scale (Wechsler Intelligence Scale for Children, fifth edition), was used to measure the cognitive abilities of children enrolled in the study. Concerning children with typical neurodevelopment, they were also included after verifying the absence of any main axis I comorbidity. To be included, they needed to present (i) ADHD-RS total raw score >19; (ii) a neurological examination in the normal range; and (iii) normal IQ.

Typically developing children (n = 24) were children of hospital staff. Inclusion criteria were children with IQs in the normal range; and children with no neurological or psychiatric abnormalities. Exclusion criteria were past or current use of psychotropic medication; and presence of visual impairment (i.e., strabismus, amblyopia etc.). The IQ in typically developing children was estimated using two subtests, assessing the verbal and the logic abilities (similarities and matrix-reasoning tests, respectively).

The investigation followed the principles of the Declaration of Helsinki, and the protocol was approved by our Institutional Human Experimentation Committee (Comité de Protection des Personnes CPP Île-de-France I). Written informed consent was obtained from the parents or legal guardians of all participants after a detailed explanation of the study procedures.

### 2.2. Eye Movement Recordings

Eye movements were recorded with the Eya Eye Tracker “https://sierra-neurovision.com/” (accessed on 25 August 2025)), a CE-approved eye-tracking device for research purposes. The EyA system benefits from cameras that capture the movements of each eye independently. The recording frequency is set at 120 Hz. Accuracy is generally 0.25 degrees. During the calibration procedure, children are invited to fix a grid of 9 points (diameter 0.5°) appearing on the screen. Each calibration point requires a fixation time of 500 ms to be validated. A five-parameter polynomial function was used to adjust the calibration data and determine the viewing angles [26]. After calibration, the fixation tasks were presented to the child. The duration of each task was short (a few seconds), allowing accurate assessment of eye movement recordings.

### 2.3. Fixation Task

**Figure 1:**
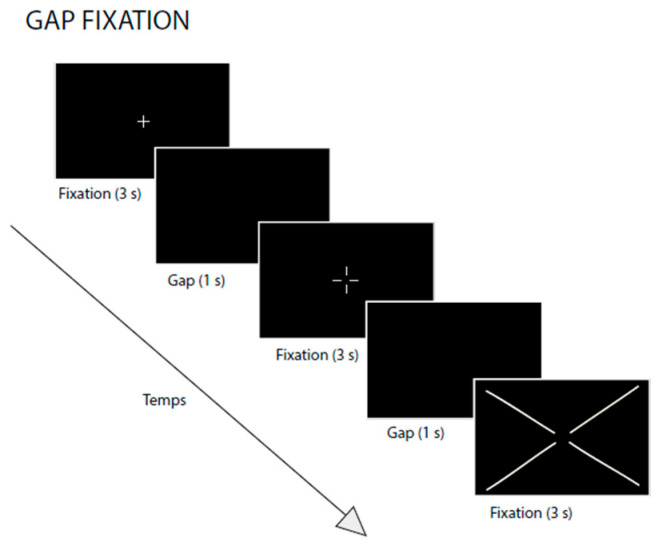
The visual sustained-fixation task using an eye-tracker. During the task, participants were instructed to fixate on a central cross for 3 s. Each fixation trial was separated by a 1 s gap period.

To assess fixation stability, participants completed a fixation task in which a target was displayed on a 15.6-inch computer monitor positioned 50 cm from their eyes. The monitor had a resolution of 1920 × 1080 pixels. Three types of different black crosses were presented on a gray background (RGB: 205, 205, 205), successively, to the participants (see Figure 1). Each visual stimulus was designed to occupy a specific visual field at a viewing distance of 50 cm. The first cross, of classic shape, had an angular size of 1° and was centered on the screen. It was displayed for 3 s (fixation condition). Following this presentation, a 1 s gap (black screen) was introduced before the second stimulus appeared. The second cross included a central interruption, leaving an empty space with a diameter equivalent to 1° at 50 cm. This stimulus was also presented for 3 s, followed by another 1 s gap. Finally, the third cross extended across the entire diagonal of the screen, but preserving a central 1° unstimulated area. This cross was also displayed for 3 s. This “gap fixation” paradigm enables us to examine visual attentional and oculomotor responses for each participant. The first target was a classic cross (1° visual angle) with a clear central intersection, allowing the child to clearly identify the position of the center. The other two targets were deliberately designed without a central intersection, leaving a 1° empty space in the center to reduce visual cues that facilitate precise fixation (see Figure 1). This progressive visual configuration was intended to maintain attention without providing a fixed reference point and to encourage the natural emergence of attentional instability in children with ADHD. The children were systematically asked to “look at the center” of each target, regardless of its appearance.

The BCEA was calculated only during the target presentation periods (3 × 3 s), and not during the gap intervals, as these did not contain any visual cues. In order to obtain a continuous and integrated measure of gaze stability, eye coordinates were analyzed in aggregate over the entire 9 s of active fixation.

A complementary study is currently underway to analyze the BCEA separately for each type of target. This approach will make it possible to precisely identify the visual conditions that cause the most difficulty for children with ADHD, and to better understand the mechanisms of instability as a function of perceptual constraints.

### 2.4. Data Analysis

The calibration factors for each eye were determined from the position of the eyes during the calibration procedure. EyA Explorer software (supplied with the eye tracker) was used to extract all eye movements from the recording. It automatically determines the start and end of each fixation, thanks to a built-in event detection algorithm. During the fixation phase, the gaze positions of the right and left eyes are recorded every 8.3 ms. This generates a point cloud for each eye, representing gaze location relative to the center of the fixation cross. An index of gaze stability is then calculated using the Bivariate Contour Ellipse Area (BCEA), with a confidence level set at 68%. The BCEA is derived from the eigenvalues λ_1_ and λ_2_ of the covariance matrix of horizontal (x) and vertical (y) eye coordinates. It is defined by the following formula, where k is a constant depending on the confidence level (k ≈ 1.14 for 68%). The eigenvalues λ_1_ and λ_2_ correspond to the variances along the principal axes of the ellipse, oriented according to the distribution of fixation points. BCEA provides an estimate of the gaze dispersion area expressed in square visual degrees (°^2^). The horizontal radius of the ellipse is proportional to the standard deviation of positions on the horizontal axis, the vertical radius to that of the vertical axis. The constant k defines the envelope of the ellipse as a function of the chosen probability [24].

BCEA = k × π × √(λ_1_ × λ_2_)

### 2.5. Statistical Analysis

Prior to conducting the main analyses, we assessed assumptions of normality and homogeneity of variance by using the Shapiro–Wilk and Levene’s tests, respectively. In order to compare clinical characteristics between the two groups of children, we used a univariate one-way ANOVA. To evaluate differences in the fixation areas, we applied a repeated-measures ANOVA with the two groups of children (ADHD and TD) as the between-subject factor and the fixation area (left vs. right eye) as within-subjects factor. We conducted all statistical analyses using JASP software (version 0.16.4.0; University of Amsterdam). Statistical significance was set at *p* < 0.05.

## 3. Results

Table 1 shows clinical characteristics of participants. The ANOVA failed to show any significant group effect for the age and Wechsler-scale (WISC-IV) scores, while it reported a significant group effect for the ADHD-RS total score (F_(1,42)_ = 823, *p* < 0.001, η^2^ = 0.95).

**Figure 2:**
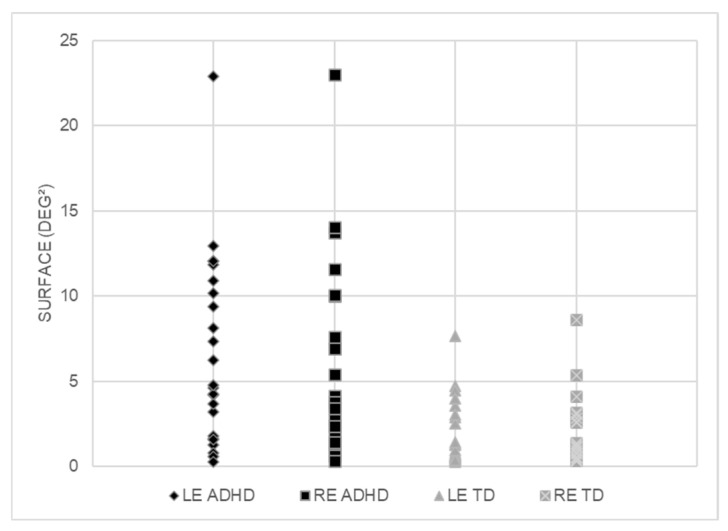
Individual values of the fixation area (deg^2^) recorded for each eye (LE = left eye and RE = right eye) for children with ADHD and TD children.

Figure 2 shows the fixation area assessed for each eye in the two groups of children tested (TD and ADHD). The ANOVA reported a significant group effect F_(1,42)_ = 11.83, *p* < 0.001, η^2^ = 0.22); the fixation area was significantly larger in children with ADHD with respect to TD children.

## 4. Discussion

The results of this study reveal significant instability in eye fixation in children with ADHD, as evidenced by an increase in the fixation surface area (BCEA) for both eyes. This measurement reflects increased variability in oculomotor control, often interpreted as the consequence of fluctuating attentional control and less effective inhibition of involuntary eye movements, two dimensions well documented in ADHD [13,14]. At the neurofunctional level, the fixation instability observed in ADHD could result from abnormalities affecting the entire network involved in inhibitory control and eye movement regulation. This network includes the frontostriato–parietal circuits [5,8,11], as well as subcortical structures such as the superior colliculus (SC) and omnipause neurons (OPN). The SC, particularly in its rostral region, contains fixation neurons that are active during gaze stabilization and participate in the inhibition of reflex saccades by modulating the activity of saccade neurons [27,28]. The OPN, located in the pontine reticular formation of the brainstem, exert tonic inhibition on saccade generators and are only briefly deactivated to allow the initiation of an eye movement [19]. Dysregulation of these neurons—for example, excessive disinhibition of the OPN or abnormal activity in the SC—could explain the frequent occurrence of intrusive saccades observed in children with ADHD. The basal ganglia, particularly the caudate nucleus and the reticular substantia nigra, play an essential role in modulating these oculomotor signals by providing downward inhibition to the SC. This control prevents involuntary movements and maintains stable fixation. Alterations in these circuits could contribute to the disinhibition observed in ADHD. Experimental data from animal models also support this hypothesis: microinjections of muscimol into the SC or SNr lead to an increase in reflex saccades and a reduction in fixation stability [19], mimicking the oculomotor profile of some children with ADHD. The persistence of this instability in the absence of visual distractors further supports the hypothesis of an intrinsic attention disorder, possibly linked to a sensorimotor integration deficit [4] or atypical maturation of visuomotor circuits, particularly the frontal eye fields and the SC [9,10]. This dysregulation appears to affect both motor and attentional spheres, reinforcing the hypothesis of a generalized inhibitory control disorder in ADHD. It could also be interpreted in light of the premotor theory of attention [29], according to which endogenous attentional orientation is based on preparatory motor activation. Attention directed toward a target would involve an inhibited saccade program, and a failure of this mechanism in ADHD could result in attentional fixation instability. At the perceptual level, micro-fixation movements play a crucial role in stabilizing the retinal image and perceptual continuity. Unstable fixation compromises visual decoding accuracy, saccade fluency, and sustained visual attention, which are essential for academic learning such as reading and writing [21,23]. This functional dimension is all the more concerning given that recent studies show that children with ADHD exhibit greater variability in performance, even in simple visual exploration tasks [30]. The results obtained are also consistent with those of Serpa et al. [31], who established reference values for BCEA in typically developing children. They showed that fixation stability improves gradually with age and that young children show increased variability. The marked increase in BCEA in children with ADHD in our study, well above the averages reported in typically developing children, confirms the specificity of this oculomotor instability in ADHD. Our work also confirms previous findings from our group, which demonstrated oculomotor impairments in children with ADHD, sometimes partially improved by pharmacological treatments [14] or following short visuo-attentional training [32]. Furthermore, the vulnerability of the attentional network in dual-task contexts, demonstrated in these children, suggests rapid cognitive overload and reduced efficiency of compensatory circuits [13]. Clinically, measuring fixation area using eye tracking and calculating BCEA is an objective, reliable, and non-invasive behavioral marker of attentional functioning. Its main advantage lies in its independence from verbal skills, motivation, or active cooperation from the child, making it a particularly suitable tool for assessing neurodevelopmental disorders. It can be easily integrated into a multidimensional approach to ADHD and paves the way for targeted interventions, such as orthoptic or visuomotor programs, whose effectiveness could be monitored quantitatively, through changes in this measure [33]. A recent case study [34] illustrates the value of this approach in the context of individualized therapeutic follow-up: in a child with ADHD treated with methylphenidate and receiving weekly orthoptic rehabilitation, a significant improvement in fixation (BCEA reduced from 2.16° to 0.7°) was observed, accompanied by a decrease in anticipatory saccades and improved eye tracking fluency. This type of protocol shows that eye tracking can be used not only to objectively assess initial attention deficits, but also to quantify progress and guide the adaptation of treatment, particularly in the context of multidisciplinary interventions. One of the specific features of our protocol is the development of a novel analysis module, based on the calculation of the fixation area, which is integrated into our software. Indeed, most eye-tracking studies use the number of intrusive saccades during a fixation task as the main indicator [2]. Although relevant, this parameter remains binary, and relatively crude: it only detects obvious breaks in fixation, and is highly dependent on technical parameters such as the detection threshold, sampling frequency (e.g., 120 Hz vs. 1000 Hz), or the software filters used. This dependence limits its sensitivity, robustness, and comparability between studies. In contrast, the BCEA-based approach provides a quantitative, continuous, and spatialized measure of gaze stability. It allows the estimation of the gaze dispersion zone around a target, thus integrating fine spatial variability, involuntary micro-displacements, subtle attentional fluctuations, post-distractor stability, and even the kinetics of return to the target after transient disorientation. This fine analysis transforms a discrete measurement into a more ecological and sensitive indicator, capable of capturing intra- and inter-individual variations that are difficult to detect using conventional markers. This approach is inspired by the work of Crossland et al. [23], initially developed in the context of microperimetry, and whose adaptation to the field of ADHD and eye tracking could represent a significant advance in the oculomotor phenotyping of attention disorders. Future work on a larger scale will be necessary to validate the reproducibility of the fixation surface in multi-center populations, identify clinical thresholds that discriminate between ADHD and other neurodevelopmental disorders, cross-reference this measurement with other oculomotor parameters (latency adn directional errors), and evaluate its evolution under the effect of pharmacological, orthoptic, or cognitive interventions.

## 5. Conclusions

This study highlights increased fixation instability in children with ADHD, as evidenced by a significantly increased fixation area. These results confirm the sensitivity of eye tracking in detecting alterations in attentional and oculomotor control. Fixation area thus appears to be a quantifiable indicator of attention dysfunction, and its integration into standardized clinical protocols could enrich the tools available for assessing and monitoring ADHD. These data also open up prospects for the development of targeted visual-attention interventions, to support traditional treatments.

Note, however, that in this exploratory study our main objective was to propose a simple method that could be applied in a clinical context, particularly for professionals working with children with ADHD. That is why we deliberately limited the analysis to an aggregated and automated measurement of the BCEA, which is easy to interpret and integrate into our analysis tool.

Finally, we would like to point out that, although BCEA is used in other fields (notably in microperimetry), its application in the context of pediatric ADHD remains very rare, and the present study contributes to validating this method as a sensitive and integrated indicator of attentional instability. We are convinced that its potential is still under-exploited, and we plan to extend its use to dynamic analyses (temporal variation, response to distractors, etc.) in our future work.

## 6. Limitations

Despite robust results, this study has several limitations. The small sample size limits the generalizability of the results. Furthermore, although the BCEA is a rigorous mathematical index of dispersion, it does not alone allow for differentiation between the mechanisms involved: distractibility, ocular impulsivity, or intrinsic attentional fluctuations.

Furthermore, the BCEA is currently used to statistically describe gaze behavior, but there is no standardized clinical classification for its interpretation in a neurodevelopmental context. Future research should evaluate the reproducibility of these measures on a larger scale, in differentiated clinical cohorts, and integrate other oculomotor parameters (saccades, latencies, and directional errors).

An important methodological point concerns the decision not to analyze the different fixation targets or transition periods (gaps) separately. Our protocol aimed to create a prolonged situation of active fixation in a deliberately unstructured visual context, in order to promote the emergence of attentional instabilities. The last two targets used were deliberately designed without an explicit central cue, with an empty space of 1° in the center, thus making the fixation task more demanding. The BCEA was calculated only during periods of visual stimulation (excluding gaps), and the data were aggregated to provide a continuous and representative measure of gaze dispersion. Although this choice limits the differential analysis by condition, it reinforces the robustness of the overall indicator used to quantify attentional instability. Our results should also be interpreted in light of the work of Thaler et al. (2013), who highlighted the influence of the shape and structure of the fixation stimulus on oculomotor stability [22]. The absence of a central cue in the last two targets likely increased the difficulty of the fixation task, thereby increasing the sensitivity of our measure to detect attentional instability. In a study currently underway, we plan to analyze gaze stability separately for each of the three targets, to determine which one poses the greatest challenge for children with ADHD. This approach will provide further insight into the interactions between visual-stimulus characteristics and oculomotor–attentional control.

Another limitation concerns the sampling frequency (120 Hz). This resolution does not allow for the detection of fine components of eye movement such as microsaccades, drifts, or tremors. These micro-movements are relevant indicators of attentional control, as shown by Hafed et al. (2011), particularly in covert attention paradigms [35]. In our study, the BCEA measurement therefore reflects an overall instability of fixation, without allowing the different underlying oculomotor components to be isolated precisely. It should also be noted that BCEA is not a method designed to detect or classify specific fixational eye movements, even under higher sampling frequencies. Rather, it provides an aggregate spatial measure of gaze dispersion over time. While high-resolution systems may give access to micro-movements, the BCEA remains a functional and global index of fixation stability, not a tool for decomposing its microstructural components. This methodological limitation restricts the detailed interpretation of the mechanisms at play, but does not alter the validity of the approach as a functional indicator of attentional stability.

## Figures and Tables

**Table 1:** Clinical characteristics (mean, SD) of the two groups of children enrolled in the study.

	ADHD Group (N = 22)	TD Group (N = 24)
Age (years)	8.75 ± 0.96	8.76 ± 1.27
ADHD-RS total score	38± 4.5	4.6 ± 1.3
WISC-V subtest scores:		
Verbal comprehension	95 ± 6	
Visual spatial	92 ± 4	
Fluid reasoning	93 ± 7	
Working memory	94 ± 8	
Processing speed	98 ± 5	
Similarities	10.9 ± 0.5	10 ± 2
Matrix reasoning	11.1 ± 2	11.5 ± 1

## Data Availability

The original contributions presented in this study are included in the article material. Further inquiries can be directed to the corresponding author(s).
